# Apportioning archaic variants among modern populations

**DOI:** 10.1098/rstb.2020.0411

**Published:** 2022-06-06

**Authors:** Kelsey E. Witt, Fernando Villanea, Elle Loughran, Xinjun Zhang, Emilia Huerta-Sanchez

**Affiliations:** ^1^ Ecology, Evolution, and Organismal Biology, Brown University, Providence, RI 02912, USA; ^2^ Center for Computational Molecular Biology, Brown University, Providence, RI 02912, USA; ^3^ Department of Anthropology, University of Colorado Boulder, Boulder, CO, USA; ^4^ Smurfit Institute of Genetics, Trinity College Dublin, Dublin, Republic of Ireland; ^5^ Department of Ecology and Evolutionary Biology, University of California, Los Angeles, CA, USA

**Keywords:** demographic history, population genetics, archaic introgression

## Abstract

The apportionment of human genetic diversity within and between populations has been measured to understand human relatedness and demographic history. Likewise, the distribution of archaic ancestry in modern populations can be leveraged to better understand the interaction between our species and its archaic relatives. Resolving the interactions between modern and archaic human populations can be difficult, as archaic variants in modern populations have been shaped by genetic drift, bottlenecks and gene flow. Here, we investigate the distribution of archaic variation in Eurasian populations. We find that archaic ancestry coverage at the individual- and population-level present distinct patterns in modern human populations: South Asians have nearly twice the number of population-unique archaic alleles compared with Europeans or East Asians, indicating that these populations experienced differing demographic and archaic admixture events. We confirm previous observations that East Asian individuals have more Neanderthal ancestry than European individuals, but surprisingly, when we compare the number of single nucleotide polymorphisms with archaic alleles found across a population, Europeans have more Neanderthal ancestry than East Asians. We compare these results to simulated models and conclude that these patterns are consistent with multiple admixture events between modern humans and Neanderthals.

This article is part of the theme issue ‘Celebrating 50 years since Lewontin's apportionment of human diversity’.

## 1. Introduction

Modern human populations vary in their patterns of genetic diversity as a result of past demographic events and interactions between populations. The serial bottlenecks that occurred as humans expanded out of Africa [1,2] and spread across the world still have a visible impact today, resulting in a continuous gradient of diversity that was influenced by human migrations originating in the African continent [2]. For example, populations that are geographically further from Africa have fewer variable sites and lower heterozygosity than populations that are geographically closer to Africa [1,3,4]. Additionally, periods of population replacement [5,6] or gene flow [7], isolation and selective pressures [8,9] have further shaped the genomes of modern populations. Allele sharing between populations is reflective of these demographic changes: many alleles are found globally while smaller numbers are found in only one or a small number of populations, usually representing novel mutations at low frequency or the result of recent gene flow between populations [10,11].

In recent decades, an additional component of human genetic diversity has been discovered and highlighted: modern human populations carry a legacy of admixture with archaic human populations, including Neanderthals and Denisovans. Neanderthal ancestry has been detected in human populations in Eurasia, Oceania and the Americas, as well as North Africans [12–14], while Denisovan ancestry has been found primarily in Asia, the Americas and Oceania [13,15–17]. Further archaic ancestry from unknown sources has even been identified in African populations [18–20]. Levels of archaic ancestry as a whole (including Neanderthal and Denisovan introgression, as well as introgression from other archaic humans in the case of Africans) vary between approximately 1% in African populations [18,19] and approximately 2% in Eurasians [13,15,16], with populations in Oceania harbouring the largest amount at approximately 6% [16]. The surviving archaic segments in modern human genomes are likely not the product of a single admixture event, but instead reflect a complex history of multiple points of contact between humans and several archaic populations [19,21–23]. Numerous demographic models and distinct introgression events have been suggested to explain the interactions between modern and archaic humans, although none has been posited that encompasses all modern human populations and archaic humans [24]. Many studies focus on Europeans and East Asians as focal populations, or on a single population of interest, such as Papuans, who have one of the highest proportions of Denisovan ancestry worldwide [16,23]. There is not yet a consensus on the number or location(s) of periods of gene flow between archaic and modern humans, and the models are continually changing as new data are being generated.

Interbreeding with archaic humans introduced novel genetic variants into modern humans, which were then shaped by demographic and selective forces. Positive [25–27] and negative [13,28–30] selection have shaped the frequency of some archaic genome segments, but genetic drift amplified by demographic processes—population contractions and expansions—along with admixture between modern human lineages are largely responsible for the current distribution of archaic variation in modern populations [31]. Gene flow from modern human populations with population-unique archaic alleles (i.e. archaic alleles that are only present in a single population) can introduce new archaic variants to a population, or gene flow from modern human populations without archaic admixture can decrease the amount of archaic ancestry in a population [32,33].

One key observation related to the distribution of archaic ancestry is that despite most Neanderthal archaeological sites being situated in western Eurasia, East Asian individuals exhibit higher Neanderthal ancestry than modern Europeans [12,13,34,35]. Some studies have suggested that differences in demographic history between East Asians and Europeans (such as a stronger bottleneck in East Asians) are sufficient to explain the elevated Neanderthal ancestry in East Asians [13,34,36]. Other studies have found that these factors explain some but not all of the difference [22,29], suggesting instead that additional Neanderthal admixture events provide a better explanation for the observed patterns in modern populations [16,22,37]. Interestingly, a study that examined the genetic differentiation between archaic ancestry segments in different populations recovered signals from two distinct Denisovan populations but only one Neanderthal population [21]. This result suggests that if Neanderthal admixture did occur more than once, it was from the same population or multiple closely related ones. Modern Europeans are also the product of multiple historic admixture and replacement events [7,38–40], and their demographic history may have affected levels of Neanderthal ancestry. The earliest Europeans, who encountered European Neanderthals, were more closely related to East Asians than modern European populations are [41] and were replaced by later migrants after all Neanderthals had become extinct [42]. Europeans further received gene flow from other Eurasian populations [32,39,43] and maintained long-term gene flow with African populations [18,44]. Because of the complexity of Eurasian demographic history, a consensus has not yet been reached as to the cause of the differences in Neanderthal ancestry between Europeans and other Eurasian populations, such as East Asians. Instead, the evidence points toward a more complex interaction of population demographic histories, natural selection and possibly multiple admixture events.

Several previous studies have inferred and quantified levels of archaic ancestry in modern human populations but have only made limited comparisons between populations. In this study we wanted to look at the distribution of archaic variation in each population to gain insight into how this variation has evolved in modern humans. Specifically, we compute the distribution and frequency of archaic variation in human populations and we quantify levels of shared and non-shared archaic variation between modern populations. We find that, similarly to non-archaic variants, the majority of Neanderthal alleles are present in multiple populations and geographical regions. Denisovan variation, however, tends to be found in one geographical region rather than being globally shared. Archaic variation in a population has also been affected by its demographic history; for example, population structure that can be identified using non-archaic variants can also be recovered using only archaic variants. We also quantify the level of archaic variation as a function of sample size, and we find that more of the Neanderthal genome can be recovered from a sample of South Asian individuals than a sample (of equal size) of Europeans or East Asians. In comparing Europeans with East Asians, we confirm that East Asian individuals harbour a larger amount of Neanderthal ancestry than European individuals, as previously reported, but more of the Neanderthal genome is recovered from a sample of multiple Europeans than an equal size sample of East Asian individuals. We use simulations to explore demographic models of archaic introgression and assess which model is most consistent with the patterns observed in the empirical data. Examining the worldwide distribution of archaic ancestry at the population level will improve our understanding of how differing demographic histories have impacted the distribution and number of archaic alleles in modern human populations.

## 2. Methods

### (a) 1000 Genome Project data

For this project, we used the 1000 Genomes Project Phase III data, which consists of low-coverage genomes (approx. 7–8× coverage) of 2504 individuals from 26 different populations [3]. These populations are categorized into five regional groups, often called ‘superpopulations’, and each has an associated three- letter acronym. African (AFR) populations include: African ancestry in the southwestern USA (ASW), African Caribbean in Barbados (ACB), Esan in Nigeria (ESN), Gambian in Western Division—Madinka (GWD), Luhya in Webuye, Kenya (LWK), Mende in Sierra Leone (MSL), and Yoruba in Ibadan, Nigeria (YRI). European (EUR) populations include: British from England and Scotland (GBR), Finnish in Finland (FIN), Iberian populations in Spain (IBS), Toscani in Italy (TSI) and Utah residents with Northern and Western European ancestry (CEU). East Asian (EAS) populations include : Chinese Dai in Xishuangbanna, China (CDX), Han Chinese in Beijing, China (CHB), Han Chinese in Southern China (CHS), Japanese in Tokyo, Japan (JPT) and Kinh in Ho Chi Minh City, Vietnam (KHV). South Asian (SAS) populations include : Bengali in Bangladesh (BEB), Gujarati Indians in Houston, Texas (GWD), Indian Telugu in the UK (ITU), Punjabi in Lahore, Pakistan (PJL) and Sri Lankan Tamil in the UK (STU). American populations (AMR) consist of admixed individuals with varying proportions of European, African and Indigenous American ancestry and include: Colombian in Medellin, Colombia (CLM), Mexican Ancestry in Los Angeles, California (MXL), Peruvian in Lima (PEL) and Puerto Rican in Puerto Rico (PUR). Throughout the paper, we will refer to the populations using the acronyms above, and any reference to a superpopulation (Africans, Europeans, East Asians, South Asians and Americans) will refer to the population sets described here. We will also use the term *Eurasians*, which refers to all populations that are part of the European, East Asian and South Asian superpopulations.

### (b) Archaic ancestry coverage

**Figure 1.**
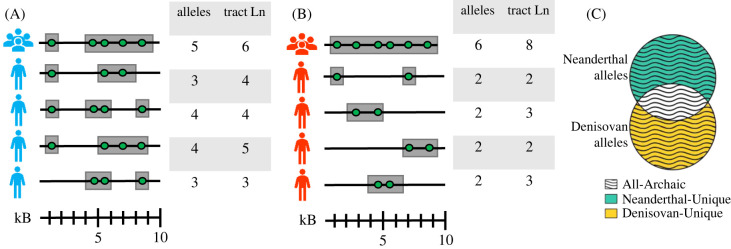
An illustration of population- and individual-level archaic ancestry coverage. Here, we show the archaic alleles (green circles) and archaic ancestry tracts (grey bars) present in a genomic region (the black line) for two populations, (A) and (B). Each population contains four individuals, and their ancestry coverage is shown next to each individual along with the total number of SNPs with archaic alleles they have as well as the archaic ancestry tract length. For the population-level coverage, each archaic allele or genome region that is found in any individual in the population counts towards the total, so population-level coverage is the sum of SNPs with archaic alleles or ancestry tracts found across all individuals in that population (the top line in A and B). (C) Illustrates the terms ‘Neanderthal-Unique’, ‘Denisovan-Unique’ and ‘All-Archaic’ as used in this paper, based on whether they are found in Neanderthals, Denisovans, or both.

To study patterns of archaic variation in modern human populations, we examined the quantity and the frequency of archaic introgressed variants. Using the autosomes, we measured the amount of archaic ancestry within a single individual as well as in a set of multiple individuals. We call this measure *archaic ancestry coverage* and use it to investigate how sample size impacts the proportion of an archaic genome recovered. We computed this quantity by using the number of single nucleotide polymorphisms (SNPs) that contain alleles that we identify as *archaic*, which is defined in §2c. Figure 1 illustrates our concept of archaic ancestry coverage at the individual and population level. Here, we show the archaic ancestry coverage in a genome region for two populations (A and B), each containing four individuals. The individual-level archaic coverage is simply the number of SNPs with archaic alleles present in each individual. For the genome region in our example, the ancestry coverage for individuals in population A ranges from 3 to 4 and the ancestry coverage for individuals in population B ranges from 1 to 2. To take the ancestry coverage of a larger number of individuals, we look for all sites where at least one individual in the sample has an archaic allele. Therefore, population A has archaic ancestry coverage of 5 and population B has archaic ancestry coverage of 6. Our example also illustrates how population- and individual-level ancestry coverage can vary between populations. Population B has higher ancestry coverage than population A at the population level, but lower ancestry coverage than population A at the individual level, suggesting that there is more archaic allele sharing between individuals within population A than between the individuals within population B. While our calculation is similar to counting the number of segregating sites (*S*), our identification of archaic sites has additional conditions. Specifically, the sites must have an allele that is also present in Neanderthals or Denisovans, while also being absent/rare in Africa (assuming introgression occurred in Eurasia). The motivation for this statistic is that the number of sites that are archaic and found primarily outside of African populations will increase as the magnitude of introgression increases. This increase in archaic alleles in populations outside of Africa is due to introgression introducing derived alleles into the recipient population and re-introducing ancestral alleles that were previously lost in the human lineage. This analysis can similarly be applied to ancestry tracts instead of alleles, provided that the ancestry tracts are identified at the individual level. In the case of ancestry tracts, the tracts for each individual can be identified using a variety of methods, such as ones that use conditional random fields [13] or hidden Markov models [45] to scan the genome for regions that have characteristics that are consistent with archaic introgression. To determine population-level archaic ancestry coverage from individual tract data, any overlapping tracts across individuals would be merged and treated as one longer archaic ancestry tract, and any part of the genome that was identified as part of an archaic ancestry tract in an individual in that population would be included in the total for that population. We would like to note that we used the tract lengths inferred and published in other studies, and we did not infer introgressed tracts ourselves.

### (c) Identifying archaic alleles and calculating ancestry coverage

We compared the 1000 Genomes (phase III) populations [46] with the Altai, Vindija and Chagyrskaya Neanderthal, and the Denisovan high-coverage genomes which were genotyped using snpAD [47,48]. Archaic genotypes were filtered with a minimum genotype quality score of 40 [8]. Alleles were considered to be ‘non-African’ if two conditions were true: (i) the allele had a frequency less than 0.01 across all African 1000 Genomes populations, and (ii) the allele had a frequency greater than 0.01 in at least one non-African population. These two conditions were set to identify sites with alleles that most likely arose outside of Africa. In addition, if the allele was also found in at least one of the sequenced archaic genomes, then we call it an archaic allele, to represent sites with alleles that were likely introgressed from archaic humans. Archaic alleles with a frequency of less than 0.01 in a population were not considered, as the frequency was below the maximum archaic allele frequency cutoff we allowed in Africans. We will refer to this method as the *SNP counting method*. The non-African alleles that were not archaic were defined as *Modern non-African* alleles, which have the same allele frequency requirements as the archaic datasets but are not shared with archaic individuals. We considered three sets of archaic alleles: *All-Archaic* (found in any of the archaic genomes), *Denisovan-Unique* (found in the Denisovans but not Neanderthals), and *Neanderthal-Unique* (found in the Altai, Chagyrskaya or Vindija Neanderthals but not Denisovans).

**Figure 2.**
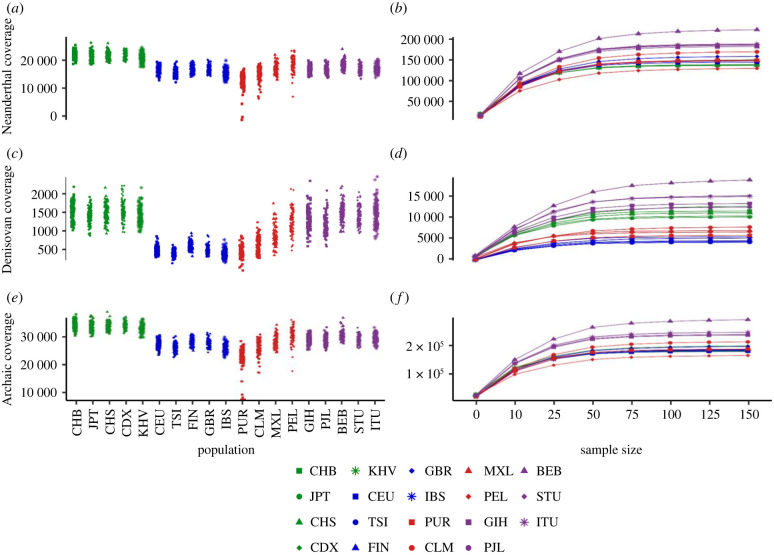
Individual archaic ancestry coverage counts for Neanderthal-Unique (*a*), Denisovan-Unique (*c*) and All-Archaic (*e*) alleles in the 1000 Genomes populations in East Asia (green), Europe (blue), the Americas (red) and South Asia (purple), and mean values for ancestry coverage of each population at varying sample sizes (*n* = 1, 10, 25, 50, 75, 100, 125, 150) for SNPs with Neanderthal-Unique (*b*), Denisovan-Unique (*d*) and All-Archaic (*f*) alleles. The ancestry coverage values for *n* = 1 on plots *b*, *d* and *f* are the median values for each population in plots *a*, *c* and *e*. Populations are colour-coded by region, and abbreviations follow standard conventions established for the 1000 Genomes Project data.

We calculated the archaic ancestry genome coverage per individual by summing up the total number of SNPs with archaic alleles in each individual's genome (figure 2*a,c,e*). We computed the archaic ancestry genome coverage in samples of randomly selected individuals from each population of varying sample size (*n* = 1, 10, 25, 50, 75, 100, 125, 150; figure 2*b,d,f*). We excluded two populations—ACB (African Caribbeans in Barbados) and ASW (African ancestry in southwest USA)—from our analyses because they contain a high proportion of African ancestry [49], so we expect them to have low levels of Neanderthal or Denisovan ancestry. Additionally, as the SNP counting method only considers alleles with a low frequency in African populations, the high proportion of African ancestry would be a confounding factor for identifying archaic alleles using our method. Given that Papuans have a higher proportion of Denisovan ancestry than the populations in the 1000 Genomes project [16,23], we also analysed archaic ancestry coverage and archaic allele sharing in Papuans (see electronic supplementary material, methods).

### (d) Simulations

**Figure 3.**
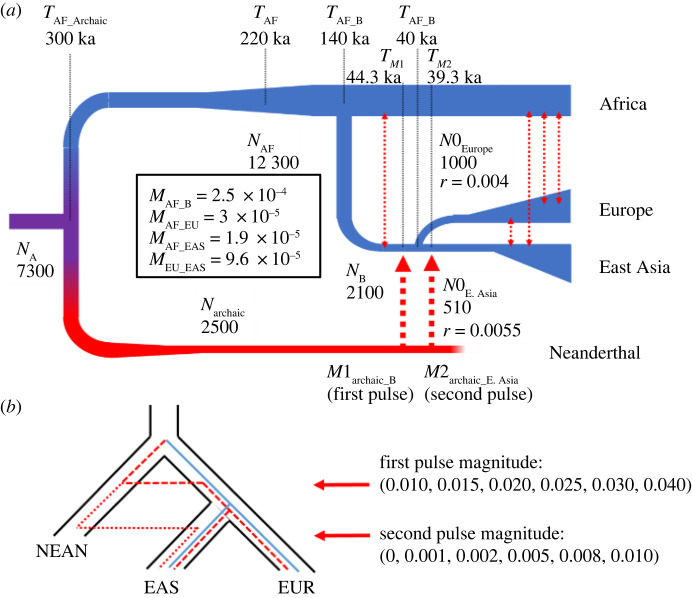
The demographic model used in our simulations and the comparison of archaic ancestry coverage in East Asians (EAS) and Europeans (EUR) between simulated and empirical data. Two pulses of introgression are depicted in this figure, but some simulations only assumed one pulse into the ancestral population of Europeans and East Asians. (*a*) A summary of the model, including population sizes and the timing of population splits and occurrences of gene flow. (*b*) An illustration of the two pulses of gene flow, with the values used for each of the demographic simulations.

We performed coalescent simulations [50] under the demographic model described below to (i) measure the power of the SNP counting method to detect archaic variants and (ii) check how demographic factors affect the number of archaic alleles. We also employed forwards-in-time simulations [51] to investigate how often alleles that are lost in Africans are called as archaic alleles, because our method uses a low frequency/absence in African populations to identify alleles as archaic instead of shared ancestral variation. A detailed description is provided in electronic supplementary material, methods. The demographic model assumed for all simulations is described in the next section and depicted in figure 3.

We also used coalescent simulations with one or two pulses of Neanderthal introgression to measure its effect on the amount of Neanderthal ancestry recovered in Europeans and East Asians as a function of sample size. These simulations are described in §2h ‘Simulating demographic models of archaic introgression'.

### (e) Demographic model

All of the simulations were conducted using the demographic model depicted in figure 3. This model is similar to the demographic model used by Villanea & Schraiber [22] (see also [16,37]), although the magnitude of introgression pulses was varied based on the simulation. This model also uses demographic parameters inferred in Gravel *et al*. [52,53], which includes the effective population sizes, split times between populations (Yorubans, Europeans and East Asians) and migration between populations. The split time between Neanderthals and modern human populations is set to 300 ka. Although there are two pulses of Neanderthal introgression in the depiction of the model, for some simulations we only had the ‘first pulse’: introgression from Neanderthals into the ancestral population of East Asians and Europeans, which was set to 44.3 ka. For the other simulations, we added a ‘second pulse’ into East Asians, which happens 39.3 ka. All fixed parameters are listed in figure 3. For different simulations we ran, some parameters were varied, which will be specified in the description of each analysis. We tested our SNP counting method to assess whether changes in the demographic model impacted archaic allele detection, and detailed methods for our benchmarking simulations can be found in electronic supplementary material, methods.

### (f) Archaic allele sharing and frequency

To investigate archaic allele sharing between populations and geographical regions (Europe, East Asia, South Asia and the Americas), for each population we identified all genome positions where at least one individual in the population had at least one copy of the archaic allele (which was at less than 1% frequency in Africans and at least 1% frequency in a non-African population). We also combined population lists in their respective regions to create region-specific position lists. We then compared those position lists to identify alleles that were unique to a population or region or were shared between multiple populations or regions and repeated this analysis for All-Archaic, Neanderthal-Unique and Denisovan-Unique allele sets (as defined in §2c; figure 1*c*).

In addition to counting all SNPs with non-African alleles present in each population and partitioning them as modern or archaic (see definitions in §2b), we also computed the allele frequency of these SNPs. The idea here is that any new genetic variants that accumulated outside of Africa were due to new mutations (represented by the *Modern non-African* set) or acquired through introgression with archaic humans (represented by the *Archaic* set). As human demographic history would affect both *Modern non-African* and *Archaic* variants similarly, we wanted to compare the frequency distribution of these variants. For each of these modern and archaic alleles, we calculated the allele frequency and classified them as ‘rare’ (0.01 < *f* < 0.2) or ‘common’ (*f* ≥ 0.2). Archaic alleles with a frequency of less than 1% in any population were not considered. We computed the ratio of common to rare alleles for *Modern non-African* and *Archaic* variants, respectively, plotted in electronic supplementary material, figure S1.

### (g) Principal component analysis

**Figure 4.**
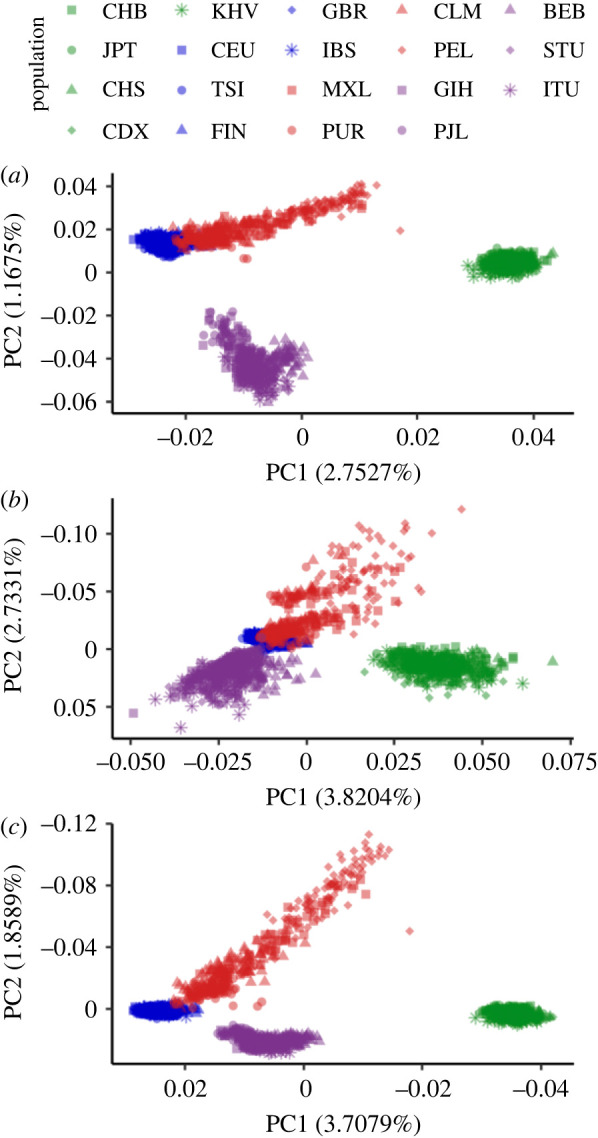
A PCA of 1000 Genomes populations, using archaic alleles with a frequency of at least 5% in one non-African population for SNPs with (*a*) All-Archaic alleles, (*b*) Denisovan-Unique alleles and (*c*) a set of SNPs with non-archaic alleles that are size-matched to the number of SNPs with archaic alleles. The non-archaic alleles are also at a frequency of less than 0.01 in Africans and have a frequency of at least 5% in one non-African population. Individuals are colour-coded by their superpopulation and distinguished by their shape.

To determine if archaic variants in humans can be used to reproduce known patterns of human population structure, we used principal component analysis (PCA). We used the archaic alleles (see definition in §2b) with a minimum frequency of 0.05 in at least one non-African population for the PCA (*n* = approximately 250 000). We used a higher minimum frequency cutoff than the 1% cutoff used for other analyses to help control for biases associated with the inclusion of low-frequency alleles in PCA calculations [54]. We also selected an equal number of randomly selected *Modern non-African* alleles (which had a frequency in Africans of less than 0.01 and a minimum frequency of 0.05 in a non-African population) to serve as a non-archaic comparison. PCAs were constructed for all three sets of archaic alleles (All-Archaic, Neanderthal-Unique, and Denisovan-Unique, figure 4*a,b*; electronic supplementary material, figure S2) and the randomized Modern allele subset (figure 4*c*) using Eigenstrat v. 6.0 [55,56]. The resulting PCAs were plotted in R v. 4.0.2 using ggplot2 [57,58]. To compare the random and All-Archaic plots more directly, we used a Procrustes analysis to plot the two PCAs on top of each other using the package MCMCpack v. 1.6-0 [59].

### (h) Comparing archaic ancestry coverage measures across methods

For the analysis of Neanderthal introgression into Europeans and East Asians, we computed the amount of Neanderthal-ancestry coverage by counting the number of Neanderthal variants in East Asian and European populations (SNP counting method) at the individual and population levels. To confirm that the results from the SNP counting method are similar to calculations using other published sets of archaic alleles or tracts, we also computed the archaic ancestry coverage using the introgressed tract lengths or alleles inferred in other studies [21,35,60] (electronic supplementary material, figure S3). We used a total of three datasets for comparative purposes: SNPs with archaic alleles identified in the 1000 Genomes populations using an LD-detection method called Sprime [21], the introgressed tracts detected in the 1000 Genomes populations using a hidden Markov model-based method called diCal-admix [35], and archaic introgressed tracts identified in 1000 Genomes populations using a conditional random field-based method [60]. For the studies that included allele data [21,35], we counted the SNPs with archaic alleles as identified by each of the studies that were present in the 1000 Genomes CEU, CHB and CHS populations. For the dataset that used introgressed tracts rather than alleles [60], we used the introgressed haplotypes for CHB, CHS, JPT, IBS, TSI, CEU, FIN and GBR 1000 Genomes project individuals, excluding X chromosome haplotypes. To compare Neanderthal ancestry coverage across European and East Asian superpopulations, we used introgressed tracts that were sorted for each individual into two haploid genomes as presented in Sankararaman *et al*. [60] and merged introgressed tracts between haploids in each sample using the merge function in BEDTools v. 2.26 [61] to find the total length of Neanderthal genome recovered. We took 100 replicates of each of nine sample sizes (1, 5, 10, 25, 50, 75, 100, 125, 150 haploid individuals) from each superpopulation to calculate the ratio of European to East Asian Neanderthal ancestry coverage. This metric illustrates how the amounts of European and East Asian archaic ancestry change relative to one another on the basis of sample size. This ratio will demonstrate whether the observed increase of archaic ancestry in East Asians compared with Europeans is observed at the population level as well as the individual level. We also compared homozygosity of Neanderthal introgressed tracts between European and East Asian individuals by pairing haplotypes as identified in [60] into their diploid individuals and identifying intersections between tracts on each allele for each diploid using the intersect function in BEDTools v. 2.26. We considered a tract homozygous if there was a tract on its paired allele that reciprocally overlapped it by at least a threshold percentage (40, 50, 60, 70, 75, 80, 90 or 95%; see electronic supplementary material, figure S4).

### (i) Simulating demographic models of archaic introgression

Since higher levels of Neanderthal ancestry in East Asian individuals compared with Europeans has been hypothesized to be due to more Neanderthal introgression events in East Asians than in Europeans, we investigated whether a single or two introgression events into East Asians can reproduce the observed relationship between the amount of Neanderthal ancestry recovered as a function of sample size (figures 1 and 3). We used msprime to simulate archaic introgression into modern Europeans and East Asians under the demographic model depicted in figure 3 (see §2e). In order to explore how various levels of admixture with archaic populations impact the amount of archaic variation recovered (archaic ancestry coverage) in modern populations, we tested two scenarios. In the first one, we consider a single ‘first pulse’ of Neanderthal gene flow into the ancestor of Europeans and East Asians where the admixture proportion is either 1, 1.5, 2, 2.5, 3 or 4%. In the second scenario, we add a ‘second pulse’ of Neanderthal gene flow into East Asians where the admixture proportion is either 0, 0.1, 0.2, 0.5, 0.8 or 1% following the East Asian–European split (figure 3*b*).

For each replicate, we simulated 198 European chromosomes, 206 East Asian chromosomes and 204 African chromosomes, matching the sampling available from the 1000 Genomes project panel for the CEU, CHB and YRI populations, respectively, as well as two chromosomes representing a Neanderthal genome. We simulated a 100 Mb chromosome using a mutation rate of 1.5e−8 bp per generation and a recombination rate of 1e−8 bp per generation [59]. Using the tree sequences output by msprime, we identified introgressed segments in the sampled chromosomes by asking which of the sampled chromosomes coalesced with the archaic lineage more recently than the human–archaic population split time. For each simulation replicate we computed the amount of Neanderthal ancestry recovered in the simulated European and East Asian populations as a function of the number of sampled chromosomes, and took the ratio of East Asian archaic ancestry coverage to European archaic ancestry coverage (EAS/EUR). By calculating this ratio, we could directly compare the simulated ancestry coverage relationship with the empirical differences in ancestry coverage between East Asians and Europeans as a function of sample size. Each combination of admixture parameters was simulated with 200 replicates. For each replicate, we resampled genomes 100 times for each sample size. For example, for a sample of size 1, we randomly sampled one European chromosome and one East Asian chromosome and took the ratio, and we did that 100 times and computed the mean across all replicates.

### (j) Assessing model fit to empirical data

For our empirical data comparison, we calculated the ratio of East Asian to European archaic ancestry coverage using the Neanderthal-Unique allele set across various sample sizes (*n* = 1, 10, 25, 50, 75, 100, 125, 150), resampling the data 100 times for each sample size to create a distribution of ratios (see electronic supplementary material, figure S3 ). We also compared our results with those of three previously published datasets: the archaic alleles identified using the method Sprime in Browning *et al*. [21], the archaic alleles identified using the program diCal-admix in [34] and the archaic introgressed tracts identified using a conditional random field method in Sankararaman *et al*. [60]. A comparison of the empirical archaic ancestry coverage is shown in electronic supplementary material, figure S3. For each dataset, we calculated the ratio of East Asian to European ancestry coverage at the sample sizes mentioned above (using alleles or tract lengths depending on the data), resampling 100 times for each size. Because our simulated data produced tract lengths, we chose to compare our simulated data with the inferred introgression maps from Sankararaman *et al*. [13]. We calculated the ratio of East Asian to European archaic ancestry coverage across sample sizes for each of the simulated datasets. We calculated mean squared error (MSE) to test the fit of each model to the empirical data:

$$\mathrm{MSE}\;=\frac{1}{n}\;\overset{n}{\underset{i=1}{\sum }}{\left({\mathrm{mean}}_{\mathrm{empirical}}-{\mathrm{mean}}_{\mathrm{simulation}}\right)}^{2},$$

where *n* = the number of simulation replicates for each model. We then calculated the Akaike information criterion (AIC) for the one-pulse model and two-pulse model with the lowest mean squared error to compare their efficacy using the following equation:

$$\mathrm{AIC}\;=\;N\times \log\left(\frac{\mathrm{RSS}}{N}\right)\;+\;2k,$$

where RSS is the residual sum of squares, *N* is the number of ratios that were calculated for each model (as well as the number of sample sizes) and *k* is the number of parameters that were variable in each model (1 for one pulse and 2 for two pulses).

## 3. Results

### (a) Testing our archaic allele identification method with simulations

To compute archaic ancestry coverage, we counted the number of sites with archaic alleles in a single individual or in a set of individuals (see §2). We tested this SNP counting method using simulations to estimate its power to detect alleles that were introduced as a result of archaic introgression (see electronic supplementary material, Methods). The method has a high statistical power to correctly identify archaic alleles (0.745 ± 0.04) (electronic supplementary material, figure S5). Nearly all of the false negatives were cases in which the allele was introgressed but was not present in a sampled Neanderthal genome. Our SNP counting method has no way to identify introgressed variants if they are not shared with a Neanderthal reference and therefore would not be expected to correctly identify alleles that derive from a Neanderthal but are not found in the sequenced Neanderthal genomes. When positions with alleles not shared with the sampled Neanderthal were excluded, the power was much higher (0.993 ± 0.003). We found that the statistical power was essentially identical between simulated East Asian and European populations (0.742 ± 0.04 for Eurasians and 0.748 ± 0.04 for East Asians, with an average difference between them of −0.0057 ± 0.023) and the amount of archaic introgression detected was robust to a range of bottleneck sizes and population growth rates (electronic supplementary material, figure S6). We also calculated the likelihood that an ancestral allele would be lost in Africa (but not in Eurasia or Neanderthals) and then be erroneously identified as an introgressed allele, and show that, on average, ancestral variation (false positive detections) only accounts for 4% of the detected archaic introgression calls (electronic supplementary material, figure S7). For some outlier simulations, we observe a proportion larger than 10% (see ‘%AncAsIntrogression’ in electronic supplementary material, figure S7), but that can be explained by the extremely low detected archaic introgression rate (less than 1%; electronic supplementary material, figure S8).

### (b) Distribution of archaic variation in modern human populations

As a first step, we identified all segregating sites with archaic alleles in the populations considered here, and applied a PCA to the set of All-Archaic sites (see §2) with archaic alleles at greater than 5% frequency. We find that archaic alleles recapitulate similar levels of population structure to a random sample of non-archaic sites that was size-matched to the number of sites with archaic alleles (figure 4*a,c*). Archaic alleles can be used to visually distinguish between East Asian, South Asian and European populations. The first principal component visually separates East Asians, South Asians and Europeans, while the second principal component differentiates the admixed American, European and East Asian populations from the South Asian populations. The first principal component also sorts the admixed American populations based on their proportion of European ancestry, so that individuals with higher European ancestry cluster more closely with Europeans (electronic supplementary material, figure S9) than individuals with less European ancestry. A Procrustes analysis of the two plots demonstrates that the East Asians, South Asians, and Europeans cluster together similarly on both plots, while the admixed American individuals are distributed along a steeper gradient in the random sample of sites compared with the archaic sites (electronic supplementary material, figure S10). Neanderthal-Unique sites show a similar pattern to that of All-Archaic sites (electronic supplementary material, figure S2), while Denisovan-Unique sites show less distinction between South Asians and Europeans compared with Neanderthal-Unique sites (figure 4*b*).

**Figure 5.**
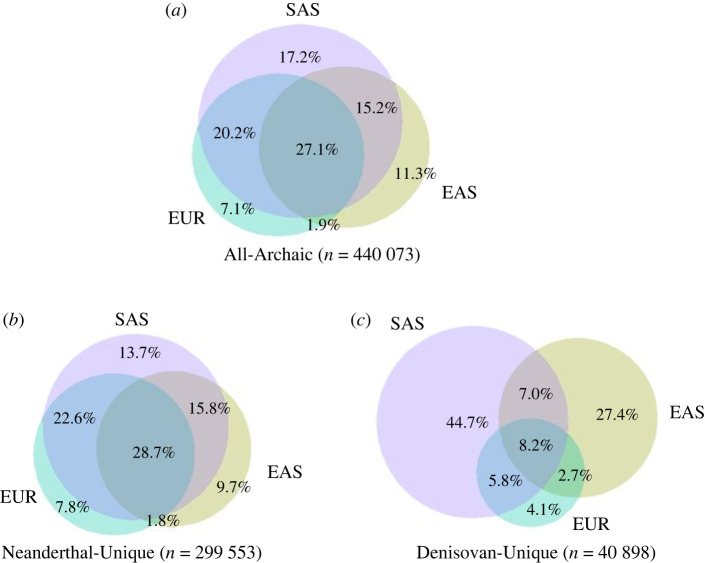
A Venn diagram showing archaic allele sharing between geographical regions in Eurasia: Europeans (EUR), East Asians (EAS) and South Asians (SAS) for (*a*) All-Archaic alleles, (*b*) Neanderthal-Unique alleles and (*c*) Denisovan-Unique alleles. The percentages refer to the percentage of alleles shared by the populations in overlapping circles.

Despite the regional differences as observed in the PCA, more archaic alleles are shared between populations and regions than are population- or region-unique (electronic supplementary material, tables S1 and S2) [62]. For example, when we examine archaic allele sharing between Eurasian populations (East Asians, South Asians, and Europeans), we find that archaic variants present in only a single Eurasian population make up 35.6% of archaic variants, representing 21.1% of South Asian alleles, 20.3% of East Asian alleles and 4.2% of European alleles (figure 5*a*). These numbers show that South Asian populations have the largest number of unique archaic alleles relative to other Eurasian populations (17.2%). If we examine only the Neanderthal-Unique alleles (figure 5*b*), the trends are similar to those observed for All-Archaic alleles (figure 5*a*). Notably, the Denisovan-Unique alleles show a different pattern, where a large proportion (approx. 72%) of Denisovan-Unique variation is private to South Asian or East Asian populations (44.7 and 27.4% respectively, figure 5*c*).

As South Asian populations harboured the largest number of unique Denisovan variants, we wanted to compare their set of archaic SNPs with those in Papuans. As Papuans have one of the highest proportions of Denisovan ancestry worldwide [16,23], we wanted to determine whether the inclusion of Papuans in the comparison would reduce the proportion of unique Denisovan variants that are private to South Asian populations. Since Papuans were not sequenced in the 1000 Genomes project, we used the 16 Papuan samples from the Simons Diversity Project [63]. When we examine archaic allele sharing between Papuans and other 1000 Genomes populations (see electronic supplementary material, Methods), we find that most Papuan Neanderthal-Unique alleles (approx. 80%) are shared with other global populations, while few Papuan Denisovan-Unique alleles (approx. 18%) are found in Europe, East Asia or South Asia (electronic supplementary material, figure S11). As most of the Denisovan variation present in Papuans is private to Papuans, there is very little sharing between Papuans and East Asian populations or Papuans and South Asian populations. Therefore, we still see that the majority of Denisovan-Unique variants present in East Asian or South Asian populations are private to these populations (approx. 53 and 55%, respectively; electronic supplementary material, figure S11). This pattern of limited allele sharing between populations may be due to the fact that multiple Denisovan-like populations have admixed with modern humans [16,23].

### (c) Comparing archaic allele frequencies between populations

While most of the variation in non-Africans is a subset of what we observed in African populations, non-African populations have accrued new alleles since their expansion out of Africa. If we ask what proportion of non-African alleles (defined by our ‘non-African’ set, see §2) were actually introduced through introgression with archaic humans (i.e alleles that are also present in the sequenced archaic individuals), we find that the proportion varies between 7 and 11% depending on the population (see electronic supplementary material, table S3). The majority (88–98%) of non-African alleles, whether looking at the modern (non-archaic) or the archaic set, have allele frequencies less than 20%. The populations with the largest proportions of high-frequency (≥20%) non-archaic or archaic alleles are found in East Asian populations and Peruvians (6–12% compared with 2–6% for other populations; electronic supplementary material, table S3). For most populations, the ratio of common to rare alleles is similar regardless of whether the alleles being considered are archaic or modern (electronic supplementary material, figure S1). For example, East Asian populations have a higher common : rare ratio than European populations for both modern and archaic alleles. However, South Asian populations have a lower common : rare ratio for archaic alleles compared with their common : rare ratio for modern alleles, suggesting that they have an excess of rare archaic alleles compared with rare modern alleles. Therefore, not only do South Asian populations have more archaic variants than East Asian or European populations, but they also have the highest proportion of rare archaic alleles (electronic supplementary material, tables S1 and S2 and figure S1). Comparison with the 16 Papuans shows that Papuans have more archaic alleles at higher frequencies than the other populations sampled (electronic supplementary material, figure S12).

### (d) Variation in archaic ancestry coverage across populations

We further looked at the individual- and population-level Neanderthal and Denisovan ancestry coverage as a function of sample size. The idea was to investigate how much of the Neanderthal or Denisovan genome could be recovered from a single or more individuals. Our hypothesis was that since the proportion of introgression is reported to be higher in East Asian individuals, then we should recover more archaic ancestry from a sample of East Asian individuals than from a sample of European individuals. To test this hypothesis, we measured archaic ancestry coverage (see §2) at various sample sizes to investigate the amount of Neanderthal variants that we could recover from a set of individuals. Figure 2*a* confirms that East Asians have more Neanderthal ancestry coverage per individual compared with individuals in other populations, consistent with previous studies [12,13,35,36,64]. For Denisovan variants, East Asian individuals exhibit similar levels of coverage to South Asian individuals (figure 2*b*). When we look at the relationship between the amount of Neanderthal or Denisovan variants and sample size, we find that East Asian populations have nearly identical ancestry coverage to European populations and admixed American populations as the sample size increases, and have lower coverage than South Asian populations (figure 2*f*).

Notably, different patterns emerge when we examine the Neanderthal- and Denisovan-Unique datasets. With Neanderthal-Unique variants, we actually recover more of the Neanderthal genome from a set of European genomes than a set of East Asian genomes, which is the opposite of what we would expect from the findings at the individual level (figure 2*a,b*)*.* This pattern suggests that while Neanderthal variants in East Asian populations are found at higher frequency than in European populations, more Neanderthal variants are shared between individuals in East Asia compared with individuals in Europe (electronic supplementary material, figure S13). Indeed, East Asian individuals are more likely to be homozygous for Neanderthal ancestry than European individuals (see electronic supplementary material, figure S4). The presence of alleles that are Denisovan-Unique or shared between Neanderthals and Denisovans seems to be sufficient to mask this pattern in the All-Archaic dataset. For Denisovan-Unique variants, we recover more from a set of East Asian individuals than Europeans, which we expect given that Europeans exhibit almost no Denisovan ancestry [60]. Perhaps most surprising is that we recover the largest proportion of a Neanderthal or Denisovan genome from any set of South Asian individuals even though South Asians have individual-level ancestry coverage similar to or lower than East Asians (figure 2*c,d*). When we compare Eurasian populations with Papuans, we find that Neanderthal ancestry coverage in Papuans is similar to Eurasian populations, while Denisovan ancestry coverage is much higher in Papuans (electronic supplementary material, figure S14), which is expected since Papuans have the largest proportion of Denisovan ancestry [16,23].

### (e) Modelling Neanderthal ancestry in Europeans and East Asians

**Figure 6.**
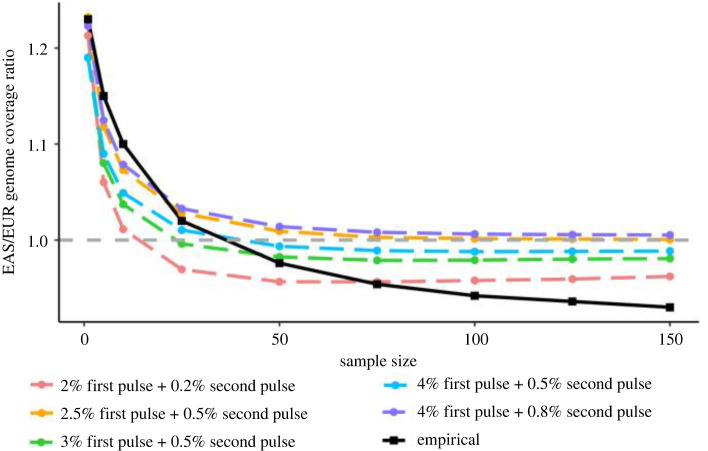
A comparison of the five best-fitting demographic models (by lowest mean squared error) to the empirical data. The *x*-axis is the number of individuals sampled to calculate ancestry coverage, and the *y*-axis is the ancestry coverage found in EAS divided by the ancestry coverage found in EUR. The dashed horizontal line denotes where the ancestry coverage would be equal across both populations. The empirical mean values (from 100 sampled replicates) are in black, and the mean values (from 100 sampled replicates each of 200 simulated datasets) of the five best-fitting models with the lowest mean squared error relative to the empirical data are shown in different colours. For all models, the ‘first’ pulse represents gene flow from Neanderthals into the ancestor of East Asians and Europeans, while the ‘second’ pulse represents archaic gene flow into East Asians specifically. The full list of models, their coverage ratio values and mean squared error are available in electronic supplementary material, table S4.

Figure 2*b* shows that, at a sample size of 25 or larger, we recover more Neanderthal ancestry from a set of Europeans than a set of East Asians. If we compare the ratio of Neanderthal-Unique ancestry coverage between East Asians and Europeans, we observe an EAS/EUR ratio of 1.2 at the individual archaic ancestry coverage level, consistent with the 20% enrichment of Neanderthal ancestry reported in the literature [12,13,35,36,64]. However, as sample size increases, the EAS/EUR ratio approaches 1.01 (figure 6), and at the highest sample sizes, Europeans actually exhibit higher archaic ancestry coverage than East Asians at the population level, with an EAS/EUR ratio of 0.97. This pattern is observed using archaic allele data, and we compared our results with introgressed segments and alleles inferred for Europeans and East Asians using alternative methods [21,35,60] (electronic supplementary material, figure S3). While each of these methods recovers different amounts of introgression for Europeans and East Asians (for example, our method recovers nearly double the number of alleles identified using the Sprime method [21]), the change in ratio with increasing sample size remains consistent.

As several studies have suggested that East Asians have more Neanderthal ancestry than Europeans owing to more than one introgression event from Neanderthals, we wanted to assess whether one or two introgression events from Neanderthals into East Asians could lead to the observed pattern. Specifically, we simulated under a demographic model that accommodates up to two introgression events from Neanderthals into East Asian populations (see Methods and figure 3*a,b*). We varied two parameters representing differing proportions of one-pulse and two-pulse introgression models, with a first pulse ranging from 1 to 4% to the ancestors of East Asians and Europeans and a second pulse from 0 to 1% to East Asians (figure 3*b*) for a total of 36 parameter combinations. We find that the parameters that minimize the mean squared error between the simulated and empirical EAS/EUR ratio curves correspond to a model with a first pulse of Neanderthal admixture of 3% and a second pulse of admixture into East Asians exclusively of 0.5% (figure 6). Interestingly, several parameter combinations capture the observed pattern of the ratio being greater than 1 at *n* = 1 and less than 1 at larger sample sizes, but none captures the exact shape of the empirical curve. The five best-fitting models have a second pulse that is 10–20% the magnitude of the first pulse, and only one of the 10 best-fitting models had only a single pulse of admixture (see electronic supplementary material, table S4). Additionally, when we use AIC to compare the best-fitting one-pulse model with the best-fitting two-pulse model, the two-pulse model has the lower score (−22.38 compared with −18.00), suggesting that a two-pulse model is more likely to accurately represent the data than a one-pulse model. The worst-fitting models were any models with two pulses of admixture where the second pulse is ≥50% of the magnitude of the first (electronic supplementary material, table S4). Single-pulse models show a similar shape to the ratio curve observed in the empirical data, but the ratio in the single-pulse models decreases more steeply with sample size, making for a poorer fit (electronic supplementary material, table S4).

## 4. Discussion

Our study of the distribution of archaic alleles and of archaic ancestry coverage at the individual- and population-levels adds a new dimension to understanding the evolution of surviving archaic variation in modern human populations. We find that archaic variants in modern human populations recapitulate the population structure that is typically observed for East Asian, South Asian, European and admixed American populations (figure 4; electronic supplementary material, figure S10). Despite this regional grouping, there is more archaic variation that is shared between populations than is population-unique (table S2), which is consistent with other studies that have explored the distribution of alleles across global populations [10,11]. The only exceptions are Denisovan-Unique variants, where the majority of SNPs with archaic alleles are unique to South Asia and to a smaller degree East Asia (figure 4*b*). This distribution of Denisovan alleles may be a consequence of contributions from distinct Denisovan populations into East and South Asians or to differences in the proportion of Denisovan introgression. Several studies have provided evidence of at least two distinct introgression events from highly diverged Denisovan-like populations in the history of modern human populations, and Denisovan ancestry of early East Asians correlates with that in present-day East Asian and Austronesian populations, but not in South Asians [16,23,65]. This differentiation between Denisovan ancestry in East Asian and South Asian populations suggests that East Asian and South Asian populations received genomic contributions from distinct Denisovan populations. Furthermore, when we include a smaller sample of Papuans, who have a much larger amount of Denisovan ancestry compared with Eurasian populations, we find that Denisovan variants in Papuans only overlap slightly with Denisovan variants in a matched-size sample of South Asians. That is, the majority of Denisovan variants in South Asians are private. This observation that most Denisovan alleles are unique to a single geographical region provides support for contributions from distinct Denisovan populations rather than a larger proportion of Denisovan introgression.

Our analysis also reveals that, unlike other populations, South Asian populations have archaic variation that tends to be at lower frequencies compared with archaic alleles in East Asian or European populations (electronic supplementary material, figure S1), as South Asians have an excess of rare archaic alleles compared with rare non-archaic alleles. Less is known about the demographic history of South Asians compared with Europeans or East Asians, but this pattern of having many archaic alleles at low frequency may be due to population structure, as it has been suggested that South Asians are descendants of multiple ancestral populations [66]. One possibility is that the ancestral populations that contributed to modern South Asian populations harboured contributions from distinct Denisovan populations and mixtures between these populations may have reduced the frequencies of Denisovan alleles in South Asians.

We also find that, consistent with previous findings, at the individual level, Neanderthal ancestry is higher in East Asian individuals than Europeans [12,13,35,36,64]. However, at the population level, Europeans have more Neanderthal alleles than East Asians, which is the opposite of our individual-level results. This reversal in the relationship between East Asian and European Neanderthal ancestry with increasing sample size suggests that East Asian populations have fewer Neanderthal introgressed segments than European populations but the segments found in East Asians are at higher frequencies, which results in higher Neanderthal ancestry coverage per individual. Conversely, European populations retain more Neanderthal segments than East Asian populations, recovering a larger portion of the Neanderthal genome at the population level (figure 2*a,b*). The retention of more unique Neanderthal variants in Europeans compared with East Asians may certainly be related to differences in population history, as East Asians experienced a more severe founder effect with a more rapid recovery than Europeans [28,34,52,53]. For instance, we find that East Asian individuals have significantly more tracts of archaic ancestry that are homozygous compared with European individuals (*p* < 0.001 for tracts with 80% overlap; electronic supplementary material, figure S4).

We used simulated datasets to test whether demographic hypotheses could explain how the ratio of Neanderthal ancestry coverage between East Asians and Europeans changes as a function of sample size. In particular, we tested the number and magnitude of Neanderthal admixture events, while also taking inferred demographic differences between East Asians and Europeans into account [53]. The parameter combinations that minimize the mean squared error correspond to a model with two pulses where the second pulse is approximately 10–20% of the magnitude of the first (figure 6; electronic supplementary material, table S4), but these parameters fail to capture the full shape of the curve. Interestingly, both single- and two-pulse models can reproduce the feature that East Asians have more archaic coverage than Europeans at an individual level, and Europeans have more coverage than East Asians as the sample size increases, suggesting that the stronger bottleneck in East Asians has a non-negligible effect on archaic ancestry retained even in the case when the actual proportion of introgression is higher in East Asians. Models where the second pulse is at least 50% of the magnitude of the first pulse result in so much archaic ancestry coverage in East Asians that the ratio remains above 1 regardless of sample size, suggesting that increasing the proportion of introgression will not result in a better fit. Single-pulse models show a similar shape to the ratio curve observed in the empirical data, but the ratio in single-pulse models decreases more steeply with sample size than the ratio in the empirical data, making for a slightly poorer fit (electronic supplementary material, table S4).

While a model with two introgression events has the smallest mean squared error and a lower AIC compared to a model with a single introgression event, none of our simple models perfectly reconstruct the EAS/EUR archaic coverage ratio curve (figure 6). While the simulated model curves all reach a consistent ratio at *n* = 50 or *n* = 100, the empirical curve continues to decrease slightly with increasing sample size, suggesting that more investigation of the demographic changes that may contribute to this pattern is needed. We acknowledge that we have only considered a small number of parameter combinations, and further exploration of the parameter space may reveal combinations of first and second pulse proportions that provide an even better fit to the data. Additionally, there are demographic models we have not considered that may also fit the empirical data more closely than a one- or two-pulse model. These demographic models include an influx of unadmixed individuals into Europe from Northern Africa creating a ‘dilution’ effect of archaic ancestry in modern Europeans [39] or the occurrence of Neanderthal admixture into Europeans as well as East Asians (a ‘three-pulse’ model). There is growing evidence of encounters between modern humans and various Neanderthal populations in geographically distinct regions of Eurasia [42,67–70]. On the question of whether Europeans also received additional Neanderthal ancestry, recent evidence indicates the earliest Europeans encountered and admixed with distinct Neanderthal lineages but failed to leave descendants in today's Europe (Oase-1 [59]), and some are more closely related to East Asian populations than modern Europeans [40]. These early Europeans were later replaced by human groups who only carried the original Neanderthal genomic ancestry shared by all Eurasians [41]. By simulating introgression models that are more complex than one or two pulses of gene flow, we may find a simulated demographic model that is an even better fit to our empirical data.

Our study highlights how examining patterns of archaic variation in modern human variation can lead to insights on the evolution of archaic variation in humans. As a case in point, we find that our examination of South Asians reveals a rich and unique pattern of archaic ancestry. Previous studies comparing archaic ancestry in Eurasians have focused mostly on East Asians and Europeans [22,28,34,65], but our results suggest that South Asians have higher archaic ancestry coverage at the population level than both Europeans and East Asians (figure 2). South Asians also display a large proportion of rare archaic alleles compared with other Eurasians (electronic supplementary material, table S3 and figure S1), and a much larger number of unique archaic alleles compared with other populations (electronic supplementary material, tables S1 and S2; figure 5). Future inclusion of other South Asian and Oceanian populations may also help characterize the dynamics of Denisovan introgression, and modelling of archaic ancestry coverage to account for periods of bottlenecks, expansions, gene flow and natural selection that followed the introgression events may reveal how evolutionary processes shaped the patterns of archaic ancestry in modern humans.

## 5. Conclusion

Inspired by Lewontin's [71] classic 1972 study, we compare archaic variation in populations from different geographical regions. We summarize archaic ancestry coverage at the individual and population level to make and test hypotheses of archaic admixture. Our analysis shows that patterns of archaic variation in South Asian populations point to complex histories of both archaic introgression and recent mixtures of multiple ancestral groups that have shaped patterns of archaic variation differently from in Europeans or East Asians. Our results also suggest that a model with a second Neanderthal introgression event into East Asians is a better fit than a model for a single pulse of introgression. Therefore, the differences in Neanderthal ancestry between East Asians and Europeans are likely not solely due to differences in recent demographic history of these populations. Closer examination of how archaic ancestry coverage patterns change under a range of demographic models with the effects of natural selection will yield a better understanding of the population history of both modern and archaic humans.

## Data Availability

1000 Genomes Phase 3 data are available at: ftp://ftp.1000genomes.ebi.ac.uk/vol1/ftp/release/20130502/. The data are provided in the electronic supplementary material [72].
